# Amygdala and Dorsal Anterior Cingulate Connectivity during an Emotional Working Memory Task in Borderline Personality Disorder Patients with Interpersonal Trauma History

**DOI:** 10.3389/fnhum.2014.00848

**Published:** 2014-10-28

**Authors:** Annegret Krause-Utz, Bernet M. Elzinga, Nicole Y. L. Oei, Christian Paret, Inga Niedtfeld, Philip Spinhoven, Martin Bohus, Christian Schmahl

**Affiliations:** ^1^Department of Psychosomatic Medicine and Psychotherapy, Central Institute of Mental Health, Mannheim, Germany; ^2^Medical Faculty Mannheim, Heidelberg University, Mannheim, Germany; ^3^Institute of Psychology, Leiden University, Leiden, Netherlands; ^4^Leiden Institute for Brain and Cognition (LIBC), Leiden, Netherlands; ^5^Addiction, Development and Psychopathology (ADAPT) Lab, Department of Psychology, University of Amsterdam, Amsterdam, Netherlands; ^6^Amsterdam Brain and Cognition (ABC), University of Amsterdam, Amsterdam, Netherlands; ^7^Department of Neuroimaging, Central Institute of Mental Health, Mannheim, Germany

**Keywords:** amygdala, anterior cingulate cortex, borderline personality disorder, emotional distraction, emotional working memory, functional connectivity, interpersonal trauma, psychophysiological interactions

## Abstract

Working memory is critically involved in ignoring emotional distraction while maintaining goal-directed behavior. Antagonistic interactions between brain regions implicated in emotion processing, e.g., amygdala, and brain regions involved in cognitive control, e.g., dorsolateral and dorsomedial prefrontal cortex (dlPFC, dmPFC), may play an important role in coping with emotional distraction. We previously reported prolonged reaction times associated with amygdala hyperreactivity during emotional distraction in interpersonally traumatized borderline personality disorder (BPD) patients compared to healthy controls (HC): Participants performed a working memory task, while neutral versus negative distractors (interpersonal scenes from the International Affective Picture System) were presented. Here, we re-analyzed data from this study using psychophysiological interaction analysis. The bilateral amygdala and bilateral dorsal anterior cingulate cortex (dACC) were defined as seed regions of interest. Whole-brain regression analyses with reaction times and self-reported increase of dissociation were performed. During emotional distraction, reduced amygdala connectivity with clusters in the left dorsolateral and ventrolateral PFC was observed in the whole group. Compared to HC, BPD patients showed a stronger coupling of both seeds with a cluster in the right dmPFC and stronger positive amygdala connectivity with bilateral (para)hippocampus. Patients further demonstrated stronger positive dACC connectivity with left posterior cingulate, insula, and frontoparietal regions during emotional distraction. Reaction times positively predicted amygdala connectivity with right dmPFC and (para)hippocampus, while dissociation positively predicted amygdala connectivity with right ACC during emotional distraction in patients. Our findings suggest increased attention to task-irrelevant (emotional) social information during a working memory task in interpersonally traumatized patients with BPD.

## Introduction

Emotional stimuli tend to capture attention because of their potential relevance to survival (Drevets and Raichle, 1998). Coping with emotional distraction (e.g., irrelevant context information, recollection of unpleasant memories) is crucial to goal-directed behavior across different life domains and has been closely related to self-control and emotion regulation (Ochsner and Gross, 2005; Rueda et al., 2005). Working memory is critically involved in the ability to ignore emotional information while maintaining goal-directed behavior, e.g., while keeping task-relevant information in mind (Banich et al., 2009; Schweizer et al., 2013).

A well-established paradigm that can be used to investigate the ability to ignore emotional distraction is the “Emotional Working Memory Task” (EWMT). In this delayed-response working memory task, participants have to keep specific information in mind (e.g., a set of human faces or a set of letters) across a short time interval. During this delay interval, either neutral or emotional distracters (e.g., pictures from the International Affective Picture System, IAPS, Lang et al., 2005) are presented. After the delay interval, participants have to indicate whether a specific stimulus (e.g., a face or a letter) was part of the initial set or not. Participants are instructed to ignore distractors and to respond as fast and accurately as possible to the probes. Prolonged reaction times and impaired accuracy after emotional distraction suggest an increased susceptibility to distraction (for a review see e.g., Iordan et al., 2013).

In previous studies that applied this paradigm in non-clinical samples, working memory impairments during emotional distraction were associated with *increased* activity in ventral brain areas including the amygdala, insula, and inferior frontal gyrus, and *decreased* activity in dorsal brain regions including parts of the dorsolateral prefrontal cortex (dlPFC), dorsomedial prefrontal cortex (dmPFC), and dorsal anterior cingulate cortex (dACC) (Perlstein et al., 2002; Dolcos and McCarthy, 2006; Anticevic et al., 2010; Chuah et al., 2010; Denkova et al., 2010; Oei et al., 2012; Iordan et al., 2013). Although the neural correlates underlying emotional distraction remain elusive, the above-mentioned studies suggest an antagonistic relationship between brain regions implicated in emotion processing (e.g., amygdala) and brain areas involved in cognitive control and working memory (e.g., dACC, dlPFC, dmPFC) (see Iordan et al., 2013). The amygdala plays a central role in emotion processing and in the initiation of stress responses (Davis and Whalen, 2001; Phan et al., 2002; Phillips et al., 2003; Ochsner and Gross, 2007; Stein et al., 2007; Ochsner et al., 2012). The dorsal proportion of the ACC (dACC) has been discussed as a region critically involved in salience detection, attention regulation, and cognitive control (Bush et al., 2000; Wager and Smith, 2003; Dosenbach et al., 2006; Weissman et al., 2006; Nee et al., 2007; Seeley et al., 2007; Menon and Uddin, 2010; Etkin et al., 2011; Menon, 2011; Niendam et al., 2012; Petersen and Posner, 2012; Clarke and Johnstone, 2013).

There is growing evidence for dynamic interactions between “hot” (“affective”) brain regions and “cold” (“executive”) brain regions during tasks that involve both affective and cognitive processing (Pessoa, 2008). Psychophysiological interaction (PPI) analysis can be used to investigate changes in the co-activation of a brain region of interest (the “seed” region) and other regions across the brain dependent on an experimental condition (Friston et al., 1997; O’Reilly et al., 2012). The principle underlying PPI is that if two brain areas are interacting in a task-dependent manner, time courses of activity in these areas will be correlated. Stronger correlations, i.e., connectivity between the seed and a “coupled” brain area is assumed to reflect an increased exchange of information between these brain areas, while no causal conclusions can be made (i.e., whether the interaction is “driven” by the seed region or the other brain area) (Friston et al., 1997; O’Reilly et al., 2012).

Dolcos et al. (2006) investigated amygdala connectivity during performance of an EWMT in a non-clinical sample. Stronger positive amygdala connectivity with inferior frontal gyrus was observed during presentation of negative distractors (IAPS pictures), and this was associated with successful behavioral inhibition of distractors (Dolcos et al., 2006). In a study by Mitchell et al. (2008), amygdala activity was positively correlated with activity in cingulate gyrus, posterior cingulate, and middle temporal cortex, while it was negatively correlated with activity in dorsolateral and dorsomedial prefrontal regions (superior frontal gyrus, middle frontal gyrus), as well as parietal regions when emotional distracters (positive and negative IAPS pictures) interfered with a cognitive task (a shape identification task). Anticevic et al. (2010) reported stronger negative correlations between amygdala activity and activity in dlPFC, dACC, anterior PFC, and frontal operculum during presentation of negative distractors (IAPS pictures) compared to neutral distractors, as well as compared to resting state in a non-clinical group (Anticevic et al., 2010). While the effects of emotional stimuli on *working memory* have been linked to negative correlations between amygdala and dorsal prefrontal regions, an enhancing effect of emotions on other memory systems such as better encoding or retrieval of self-relevant emotional events has been associated with increased co-activation in the amygdala and medial temporal lobe regions including hippocampus and parahippocampal gyrus (for a review see e.g., Dolcos et al., 2012).

The ability to voluntarily modulate responses to emotional information through the use of cognitive strategies, e.g., through shifting attention away from irrelevant or unwanted emotional material, is a crucial part of cognitive emotion regulation (Ochsner and Gross, 2005; Banks et al., 2007; Ochsner and Gross, 2007; Schweizer et al., 2013). This ability seems to be impaired in stress-related psychiatric disorders such as borderline personality disorder (BPD) and (complex) Posttraumatic Stress Disorder (PTSD). Key features of these disorders include difficulties in discriminating between harmless and threatening cues, affective hyperreactivity, pronounced deficits in emotion downregulation, and traumatic re-experiencing (e.g., intrusions) (Elzinga and Bremner, 2002; Lieb et al., 2004; Banich et al., 2009; Schweizer and Dalgleish, 2011; Ford and Courtois, 2014; Schmahl et al., 2014). Intrusive memories of past traumatic events can be spontaneously triggered by traumatic reminders and are usually accompanied by strong sensory impressions, as if the event was happening right now (Ehlers et al., 2004; Ford and Courtois, 2014). Emotional distress caused by traumatic reminders can interfere with goal-directed behavior in everyday life, which can have detrimental effects on various life domains from social interactions to academic success (Ford and Courtois, 2014).

In previous studies that used the EWMT, patients with BPD showed prolonged reaction times associated with increased amygdala activity during emotional distraction (presentation of negative IAPS pictures) compared to healthy controls (HC) (Krause-Utz et al., 2012; Prehn et al., 2013; Krause-Utz et al., 2014a). Studies that applied similar paradigms observed a failure to activate the ACC (Wingenfeld et al., 2009: Emotional Stroop Task; Silbersweig et al., 2007: Emotional GoNoGo Task) or increased ACC activation (Holtmann et al., 2013: Emotional Flanker Task) along with amygdala hyperreactivity in BPD patients compared to healthy participants. Findings of these studies complement results of functional magnetic resonance imaging (fMRI) studies suggesting a hyperreactivity of limbic brain regions during emotional challenge in BPD patients, although there are also discrepant findings (for a review see e.g., New et al., 2012; O’Neill and Frodl, 2012; Ruocco et al., 2013; Winter et al., 2014; Krause-Utz et al., 2014b).

Apart from emotion dysregulation, dissociation is another process that may modulate emotional distractibility, i.e., amygdala activity and connectivity, in stress-related disorders such as BPD. A large proportion of individuals with BPD reports dissociative experiences (Stiglmayr et al., 2008) involving disruptions of usually integrated functions such as depersonalization, derealization, reduced sensory processing, and disturbed memory, as well as emotional detachment and numbing (American Psychiatric Association, 2013). Dissociation was suggested to represent an (over)modulaton of – otherwise overwhelming – emotions in stressful situations, possibly associated with increased frontal control in medial prefrontal regions along with dampened amygdala activation (Lanius et al., 2010). In our above-mentioned study, amygdala activity during presentation of emotional distractors (aversive interpersonal IAPS pictures) was negatively correlated with self-reported increase of state dissociation in the BPD group (Krause-Utz et al., 2012). In the same sample of interpersonally traumatized BPD patients, trait dissociation positively predicted the strength of the coupling between amygdala and dlPFC during resting state (Krause-Utz et al., 2014c).

Borderline personality disorder patients further showed diminished negative correlations between dACC and posterior cingulate (Krause-Utz et al., 2014c) as well as imbalanced inter-network connectivity (Wolf et al., 2011; Doll et al., 2013) during resting state. In the context of experimentally induced fear or threat, increased amygdala connectivity with rostral ACC (Cullen et al., 2011) and ventromedial PFC (Kamphausen et al., 2013) was found in BPD patients compared to HC. In another previous study, BPD patients showed positive amygdala connectivity with the middle frontal gyrus during an instructed emotion downregulation task when the presentation of negative IAPS pictures was combined with warmth (i.e., not painful) temperature (Niedtfeld et al., 2012). Recently, Koenigsberg et al. (2014) reported increased connectivity between insula and ventral ACC during the habituation phase, i.e., repeated presentation of negative IAPS pictures in patients with BPD compared to patients with avoidant personality disorder.

To our knowledge, no study so far has investigated amygdala and dACC connectivity during performance of the EWMT in BPD patients. Moreover, little is known about how dissociative states may modulate amygdala connectivity during emotional distraction.

Here, we re-analyzed data from our above-mentioned study in 22 unmedicated BPD patients with a history of interpersonal trauma and 22 healthy participants, who performed the EWMT during fMRI (Krause-Utz et al., 2012). The bilateral amygdala and bilateral dACC were *a priori* defined as seed regions of interest given their role in neurobiological models of affective-cognitive interactions delineated above as well as in BPD psychopathology. We used PPI to analyze task-related changes in connectivity between each seed and other areas across the brain. Based on previous research, stronger negative correlations between amygdala and dorsal frontal brain regions involved in cognitive control (dlPFC, dmPFC, dACC) were expected during emotional distraction. We further expected significant group differences, especially during presentation of negative distractors. To investigate how working memory performance (reaction times) and self-reported increase of state dissociation may predict amygdala connectivity during presentation of negative distractors additional whole-brain regression analyses were performed.

## Materials and Methods

### Sample

A total sample of 53 women [26 patients with BPD according to DSM-IV (American Psychiatric Association, 2000) and 27 HC] aged between 18 and 45 was recruited. Patients with BPD were recruited by advertisement on websites or referred from the inpatient treatment unit of the Department of Psychosomatic Medicine and Psychotherapy at the Central Institute of Mental Health (CIMH) in Mannheim, Germany. In parallel, healthy participants who matched to patients regarding age and education were referred from a pool of healthy individuals that had been recruited by newspaper advertisement and had agreed to participate in future studies of our research group. Two patients with BPD had to be excluded because of alcohol abuse. One patient and two HC canceled study participation at the beginning of the MR scan due to unexpected claustrophobia. One HC was excluded because she reported repeated self-injurious behavior in the past. Data from three HC and one patient had to be excluded from the final analysis due to movement artifacts and/or missing button presses during the EWMT.

The final sample comprised 44 women (22 BPD patients and 22 HC). All participants underwent diagnostic assessments including the Structured Interview for DSM-IV Axis-I (SCID-I, First et al., 1997) and International Personality Disorder Examination (IPDE, Loranger, 1999) by trained diagnosticians. Further clinical assessment included questionnaires on BPD symptom severity (Borderline Symptom List 95, BSL-95BSL-95, Bohus et al., 2001, 2007) and trauma history (Childhood Trauma Questionnaire, CTQ, Bernstein et al., 2003; Posttraumatic Stress Diagnostic Scale, PDS, Foa, 1995). All participants completed questionnaires on depressive symptoms (Beck Depression Inventory, BDI, Beck et al., 1961), state anxiety (State Anxiety Questionnaire, STAI-X1, Spielberger et al., 1970), and trait dissociation (Dissociative Experience Scale, DES, Bernstein and Putnam, 1986) (Krause-Utz et al., 2012). Immediately before and after the experiment, all participants further completed the Dissociation Stress Scale 4 (DSS-4) (Stiglmayr et al., 2010). The DSS-4 is a self-rating scale consisting of four items measuring current dissociative experience (depersonalization, derealization, altered hearing, and pain perception) as well as one item on current arousal (all between “0 = not at all” and “9 = extremely”) (Stiglmayr et al., 2010). General exclusion criteria were severe somatic illness and criteria related to MRI (metal implants, left-handedness, claustrophobia, and pregnancy). All patients with BPD were free of medication and did not abuse alcohol or other substances within the last 6 months. Further exclusion criteria were current major depression, lifetime psychotic disorder, bipolar affective disorder, mental retardation, developmental disorder, and a life-threatening suicidal crisis. Exclusion criteria for the HC group were a lifetime history of psychiatric disorders, as well as trauma. The control group consisted of 22 HC. The patient sample consisted of 22 women meeting criteria for BPD according to DSM-IV (American Psychiatric Association, 2000). All patients fulfilled the DSM-IV criterion for affective instability, and all patients reported a history of interpersonal traumatization including emotional maltreatment (e.g., neglect, emotional abuse), physical abuse, and/or sexual abuse as assessed by the CTQ and PDS. Nine patients (~41%) currently met diagnosis of PTSD. Descriptive statistics of demographic variables and questionnaires are reported in Table 1. There were no significant group differences in age, years of education, and body mass index (BMI) (Table 1).

**Table 1:** **Demographic and clinical variables in healthy controls (HC) and patients with Borderline personality disorder (BPD_D) and results of the univariate analysis of variance (ANOVA)**.

	HC (*n* = 22)	BPD (*n* = 22)	*t*-tests (df = 42)
Age (in years)	27.41 ± 8.49	28.18 ± 7.02	*t* = 0.33; *p* = 0.744
Body mass index	23.24 ± 4.00	25.45 ± 6.69	*t* = 1.31; *p* = 0.197
Years of education	12.14 ± 1.46	11.73 ± 1.49	*t* = 0.92; *p* = 0.362
DSS-4 before fMRI	0.10 ± 0.20	1.97 ± 1.73	*t* = 4.91, *t* = 5.75; all *p* < 0.001
DSS-4 after fMRI	0.13 ± 0.26	2.97 ± 2.25	
DES	2.45 ± 1.89	30.85 ± 15.27	*t* = 8.66; *p* = < 0.001
BSL-95 (mean)	0.24 ± 0.11	1.92 ± 0.57	*t* = 13.48; *p* = < 0.001
STAI	34.10 ± 9.04	50.16 ± 8.32	*t* = 6.14; *p* = < 0.001
BDI	1.34 ± 1.74	23.86 ± 9.91	*t* = 10.50; *p* = < 0.001
**Comorbidities**
PTSD current	*n* = 0	*n* = 9 (~41%)	
MD lifetime	*n* = 0	*n* = 8 (~36%)	
Social phobia (current)	*n* = 0	*n* = 6 (~27%)	
Specific phobia (current)	*n* = 0	*n* = 2 (~9%)	
Panic disorder (current)	*n* = 0	*n* = 6 (~27%)	
GAD (current)	*n* = 0	*n* = 3 (~13%)	
Bulimia nervosa (current)	*n* = 0	*n* = 6 (~27%)	
Anorexia nervosa (current)	*n* = 0	*n* = 7 (~31%)	
OCD (current)	*n* = 0	*n* = 4 (~18%)	

**n*, number of participants; PTSD, posttraumatic stress disorder; MD, major depressive disorder; GAD, generalized anxiety disorder; OCD, obsessive-compulsive disorder. Data from questionnaires are presented in mean score ± standard deviation*.

### Emotional working memory task

**Figure 1:**
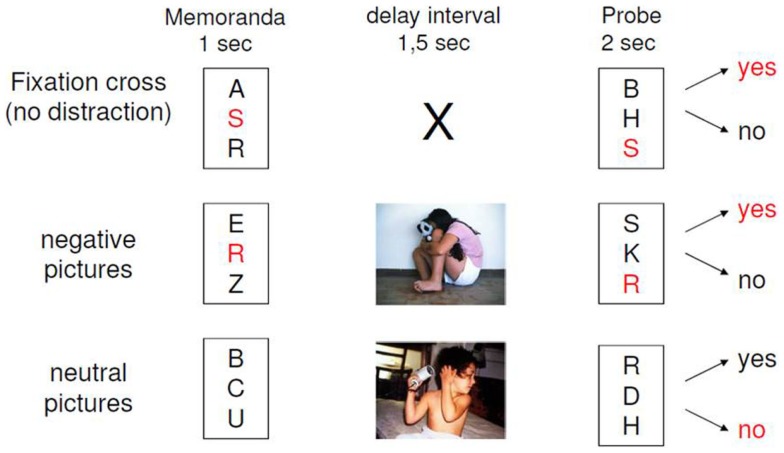
**Design of the emotional working memory task (EWMT)**.

The experimental design of our paradigm is depicted in Figure 1. The EWMT was an adapted Sternberg item recognition task (Sternberg, 1966), modified by Oei and colleagues (Oei et al., 2009; Oei et al., 2010; Oei et al., 2012; Krause-Utz et al., 2012; Krause-Utz et al., 2014a). The present version consisted of 48 trials, each starting with the presentation of a set with three uppercase letters (memoranda, 1000 ms). After a delay interval (1500 ms), again a set of three uppercase letters was presented (probe, 2000 ms). Participants had to press the “yes” or “no” button indicating whether they had recognized a target or not. In half of the trials, one of the three memoranda was present in the probe. During the delay interval, either no distractors (only a fixation cross) or neutral distractors versus negative distractors were presented. Distractors were pictures from the IAPS, which were selected based on arousal and valance ratings in the general population (Lang et al., 2005). Negatively arousing IAPS depicted interpersonal scenes of interpersonal violence (e.g., a sexual attack, physical assault, a beaten and neglected child, or a physically mutilated body). Neutral pictures were matched to negative pictures with regard to number of persons and complexity of the scene in order to avoid confounding differences in visual information processing. This means that neutral distracters were IAPS pictures, which depicted naturalistic interpersonal scenes (e.g., people at a market place or people in a supermarket), which had been rated as neutral (according to valence and arousal ratings) in the general population (Lang et al., 2005). Target-present and target-absent trials were equal in both conditions. The presentation of the conditions within the EWMT was balanced in a pseudo-random manner. In addition to the 3 conditions of the EWMT, 15 trials of the Sternberg item recognition task without distraction (i.e., only a fixation cross) were presented at the beginning of the scan as a measure of baseline working memory. Software Presentation (Neurobehavioral systems http://www.neurobs.com/) was used to present stimuli and record behavioral data. After scanning, participants rated the pictures together with 30 foils (similar IAPS pictures) regarding arousal and distraction (difficulty of shifting away attention from the picture) as perceived during the task (between “0 = not at all” and “9 = extremely”) and *post hoc* recognition of the pictures was tested.

As previously reported, we found that this paradigm was capable of inducing emotional distraction in terms of slower reaction times (Krause-Utz et al., 2012; Krause-Utz et al., 2014a) and increased activity of the amygdala compared to distraction by neutral pictures.

### Procedure

The experiment was approved by the local ethics committee (University of Heidelberg, in accordance to the World Medical Association’s Declaration of Helsinki) and took place at the CIMH in Mannheim, Germany. All participants received information about the experiment and scanning procedure and written informed consent was obtained. At the beginning of the study, participants underwent diagnostics (SCID-I, IPDE) and basic clinical assessment as described above, and practiced the EWMT outside the scanner. Immediately before and after scanning, levels of acute dissociation were assessed by the DSS-4. Inside the scanner, participants performed the EWMT, while gradient echo planar imaging (EPI) sequences were acquired (event-related fMRI). Participants were instructed to focus on the middle of the screen, concentrating only on the EWMT and ignoring the distracting pictures. To ensure that participants understood the instruction correctly, they practiced the EWMT outside the scanner and were given feedback by the experimenter (see above). At the end of the experiment, participants were thanked, debriefed, and paid for their participation.

### Scanning protocol

Scanning was conducted by a Siemens TRIO-3T MRI (Siemens Medical Solutions, Erlangen, Germany). Using 3-D magnetization prepared rapid acquisition gradient echo (T1-weighted contrast, voxel size 1 mm × 1 mm × 1 mm), a high-resolution anatomical scan was acquired for each participant as an individual template for the functional data. Using event-related fMRI (Friston et al., 1998), T2-weighted EPI for measurement of BOLD signal [field of view = 210 mm × 210mm, voxel size = 3 mm × 3 mm × 3 mm, echo time = 30 ms, TR = 2500 ms] with 40 contiguous 3 mm sagittal slices in a 64 × 64 matrix was used. The first five scans were discarded to minimize T1 effects. Head movement artifacts and scanning noise were restricted using head cushions and headphones within the scanner coil.

### Statistical analysis

Analysis of the behavioral data (working memory performance, picture ratings, and *post hoc* recognition of the pictures) were previously reported (Krause-Utz et al., 2012).

Functional imaging data were analyzed using standard procedures implemented in the Statistical Parametric Mapping package 8 (SPM8; Neurobehavioral systems, Berkeley, CA; http://www.fil.ion.ucl.ac.uk/spm/). Preprocessing of the EPI time series included slice time correction, spatial realignment, and unwarping to correct for head motion, co-registration onto participants’ high-resolution T1 scan, normalization to the standard brain of the Montreal Neurological Institute (MNI) space, and smoothing using a Gaussian kernel with a full width at half maximum (FWHM) of 9 mm.

The statistical analyses of our event-related design relied upon the general linear model to model effects of interest (Friston et al., 1995) as implemented in SPPM8. For each participant, task-related activity was identified by convolving a vector of the onset times of the following two experimental events of interest with a canonical hemodynamic response: (1) “neutral distracters” (IAPS pictures) and (2) “negative distracters” (IAPS pictures). We further defined the following events as regressors of no interest: (i) no distraction during the delay interval of the task, (ii) memoranda (target letters), and (iii) probes. The GLM further included nuisance variables to control for movement artifacts.

#### Psychophysiological interaction analysis

We used PPI to analyze changes in connectivity between a seed region of interest and other brain regions dependent on an experimental condition (psychological component). Using PPI, brain regions across the whole brain can be identified whose time courses are significantly correlated to time courses of the seed region given an experimental condition. Thereby, it is possible to analyze whether brain regions are more strongly correlated in one experimental condition than in the other or in one group compared to the other. Increased connectivity (i.e., correlation) of brain regions is assumed to reflect an increased exchange of information between these brain areas (Friston et al., 1997; O’Reilly et al., 2012), while the causality of this direction remains unknown (i.e., which brain area drives the interaction).

In our PPI analysis, two seed regions of interest were *a priori* defined based on models of affective–cognitive interactions and previous research in BPD (as delineated above): (1) bilateral amygdala and (2) bilateral dACC. Since the amygdala is a small structure, an anatomical mask of the bilateral amygdala was created based on the Automatic Anatomical Labeling (AAL) software as provided in SPM8. For the bilateral dACC, a sphere of 9 mm was created around a pre-defined voxel (MNI coordinates *X* = 5, *Y*  = 19, *Z* = 28) as reported in previous studies in a non-clinical sample (seed “I4” in Margulies et al., 2007) and in patients with BPD (Krause-Utz et al., 2014c).

For each participant, the mean time series of activity in each region of interest were extracted from the voxels falling within each mask.

The design matrix (general linear model) of our first-level analysis contained three columns: (1) the “psychological variable” (i.e., experimental condition of interest), (2) the time series of activation in the seed region, and (3) the interaction of both. The regression coefficient modeling the interaction term of the psychological variable and the time course of activation in the seed region (“PPI regressor”) provides a measure for connectivity identifying brain regions whose time courses of activity are significantly correlated to activity in the seed dependent on an experimental condition.

Separate first-level analyses for “neutral distracters” and “negative distracters” were performed for each seed. This means, for each participant, separate PPI regressors (i.e., correlations of the seed region and other regions) for “neutral distracters” and “negative distracters” were created for the amygdala seed and the dACC seed separately. A contrast of 1 for the PPI regressor and 0 elsewhere was applied to reveal clusters showing a significant positive regression slope with activity in the seed region of interest in a task-dependent manner.

Our second-level analysis was based on our two research questions: first, we aimed to analyze task-related changes in connectivity between the seed regions and other areas across the brain as an effect of *valence*, i.e., negative distracters compared to neutral distracters. Second, we were interested in the effect of *group* on task-dependent connectivity of the seeds, particularly during presentation of negative distractors.

First-level contrasts of the PPI regressors for “neutral distracters” and “negative distracters” were fed into separate whole-brain 2 × 2 Full Factorial models for each brain region (i.e., amygdala and dACC). This means, we created two 2 × 2 Full Factorial Models comprising the factor “*Group*” (two levels: “BPD,” “HC”) and the factor “*Valence*” (two levels: “neutral distracters,” “negative distracters”) resulting in four cells. One 2 × 2 Full Factorial Model was created for the amygdala seed and the other 2 × 2 Full Factorial Model was created for the dACC seed.

In each 2 × 2 Full Factorial Model, F contrasts for the main effect of the two independent variables “*Group*” (BPD, HC) and “*Valence*” (“neutral distractors,” “negative distractors”) and their interaction were defined. To follow-up significant main effects of valence, T contrasts for neutral > negative distractors (“positive effect of valence 1”) and vice versa (negative > neutral distractors) were evaluated for the full sample within each 2 × 2 Full Factorial Model.

As this was one of our main contrasts of interest, additional between-group analyses for amygdala connectivity as well as dACC connectivity during the presentation of negative distractors were performed using independent *t*-tests on the whole-brain level (i.e., connectivity during negative distractors in BPD > HC and in HC > BPD).

In all second-level analysis, clusters were determined using a significant threshold of *p* < 0.001 uncorrected at a voxel-wise whole-brain level. Clusters exceeding a *Z*-value of >3.1 and a cluster size of *k* ≥ 10 contiguous voxels are presented.

Based on our *a priori* hypothesis of amygdala connectivity with dorsal prefrontal regions during presentation of negative distractors, small volume corrections (SVCs) were applied for amygdala connectivity with dlPFC and dmPFC regions. Anatomical masks of the dlPFC and dmPFC were created based on the AAL software as provided in SPM8. These masks were then used for SVCs of clusters determined by the main effect of valence of the 2 × 2 Full Factorial Model of amygdala connectivity as well as for clusters determined by the between-group *t*-tests for amygdala connectivity during negative distractors. Clusters revealed by SVCs are indicated (by an asterisk) in the Section “Results” (Tables S1–S3 in Supplementary Material). No SVCs were applied for all other contrasts.

#### Regression analyses

To examine whether reaction times predicted amygdala connectivity during emotional distraction, first-level contrasts of interaction terms for amygdala connectivity during “negative distractors” were entered together with reaction times (in milliseconds) into whole-brain regression analyses for the BPD group and the HC group separately.

For the BPD group, another whole-brain regression analysis with self-reported increase of dissociation as regressor of interest was performed. The mean increase of dissociation (DSS-4 scores post-experiment minus DSS-4 scores pre-experiment) was defined as regressor of interest, because we previously reported significant negative correlations between amygdala activity and mean DSS-4 increase during presentation of negative distractors in the BPD group (Krause-Utz et al., 2012). First-level contrasts of interaction terms for amygdala connectivity during presentation of “negative distractors” were entered together with mean increase of DSS-4 scores into a whole-brain regression analysis.

In all regression analyses, clusters were determined using a significance threshold of *p* < 0.001 uncorrected at a voxel-wise whole-brain level. Clusters meeting a *Z*-value of >3.1 and a cluster size of *k* ≥ 10 contiguous voxels are presented.

## Results

Behavioral data and whole-brain activation patterns during performance on the EWMT were previously reported (Krause-Utz et al., 2012). In brief, significantly prolonged reaction times during presentation of negative distractors were observed in BPD patients compared to HC. There were no significant group differences in accuracy (i.e., errors). Both BPD patients and HC showed a significant increase in amygdala activation during presentation of negative distractors. Amygdala activity during emotional distraction was significantly higher in BPD patients than in HC.

Results of our present PPI analysis are presented per seed in the following.

### Amygdala connectivity

#### Main effects and interaction effect (2 × 2 Full Factorial Model)

Complete results of the main effects and interaction effects (F contrasts) of the 2 × 2 Full Factorial Model for amygdala connectivity can be found in Table S1 in Supplementary Material.

##### Main effect of valence

**Figure 2:**
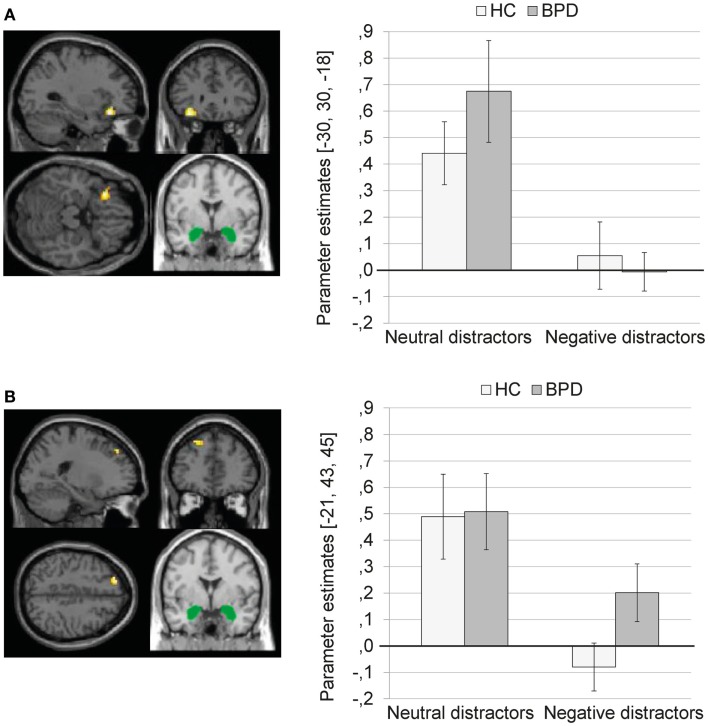
**(A)** Means ± standard errors of the mean (SEM) of parameter estimates for connectivity of the bilateral amygdala seed (depicted in green) with left inferior frontal gyrus (MNI: −30, 30, −18) during presentation of neutral distractors and negative distractors in patients with borderline personality disorder (BPD) and healthy controls (HC). **(B)** Means ± SEM of parameter estimates for amygdala connectivity with left superior frontal gyrus (MNI: −21, 43, 45) during presentation of neutral and negative distractors in BPD patients and HC.

The 2 × 2 Full Factorial revealed a significant main effect of valence on amygdala connectivity with left inferior frontal gyrus (see Figure 2A). In addition, a significant main effect of valence on amygdala connectivity with left lingual gyrus, bilateral fusiform gyrus, left parahippocampal gyrus (including parahippocampal place area, BA19), left hippocampus, right posterior cingulate, right middle temporal gyrus, and right caudate was observed.

The SVC with the dlPFC mask revealed a significant cluster in the left superior frontal gyrus (BA9) (see Figure 2B; Table S1 in Supplementary Material). The SVC with the dmPFC revealed no significant clusters.

The coupling of amygdala with the above-mentioned brain regions was significantly weaker during presentation of negative distractors than during presentation of neutral distractors (see Table S2 in Supplementary Material). The T contrast negative > neutral distractors revealed no significant clusters (see Table S2 in Supplementary Material).

##### Main effect of group

**Figure 3:**
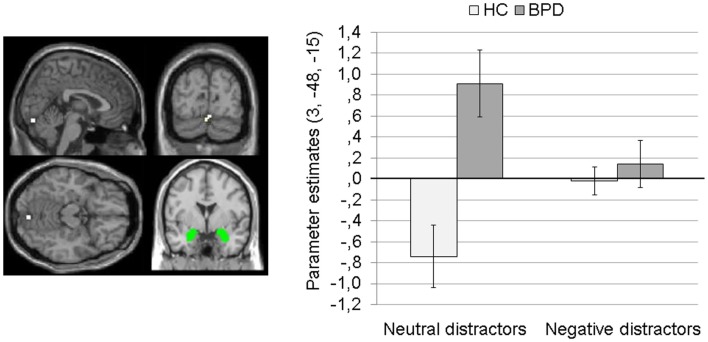
**Means ± standard errors of the mean (SEM) of parameter estimates for connectivity of the bilateral amygdala seed (depicted in green) with right lingual gyrus (MNI: 3, −84, −15) revealed by the main effect of group of the 2 × 2 Full Factorial Model for amygdala connectivity in patients with borderline personality disorder (BPD) and healthy controls (HC)**.

The 2 × 2 Full Factorial Model revealed a significant main effect of group on amygdala connectivity with a cluster in the right lingual gyrus (see Table S1 in Supplementary Material). BPD patients showed positive amygdala connectivity with right lingual gyrus during both EWMT conditions, most prominently during presentation of neutral distractors. HC showed negative amygdala connectivity with right lingual gyrus during presentation of neutral distractors and no (or only marginal) coupling during presentation of negative distractors (see Figure 3).

##### Interaction effect

There was no significant interaction effect at *p* < 0.001 (*k* ≥ 10, *Z* < 3.1).

#### Independent *t*-test for between-group differences during negative distractors

Complete results of the independent *t*-test for amygdala connectivity during presentation of negative distractors can be found in Table S3 in Supplementary Material.

**Figure 4:**
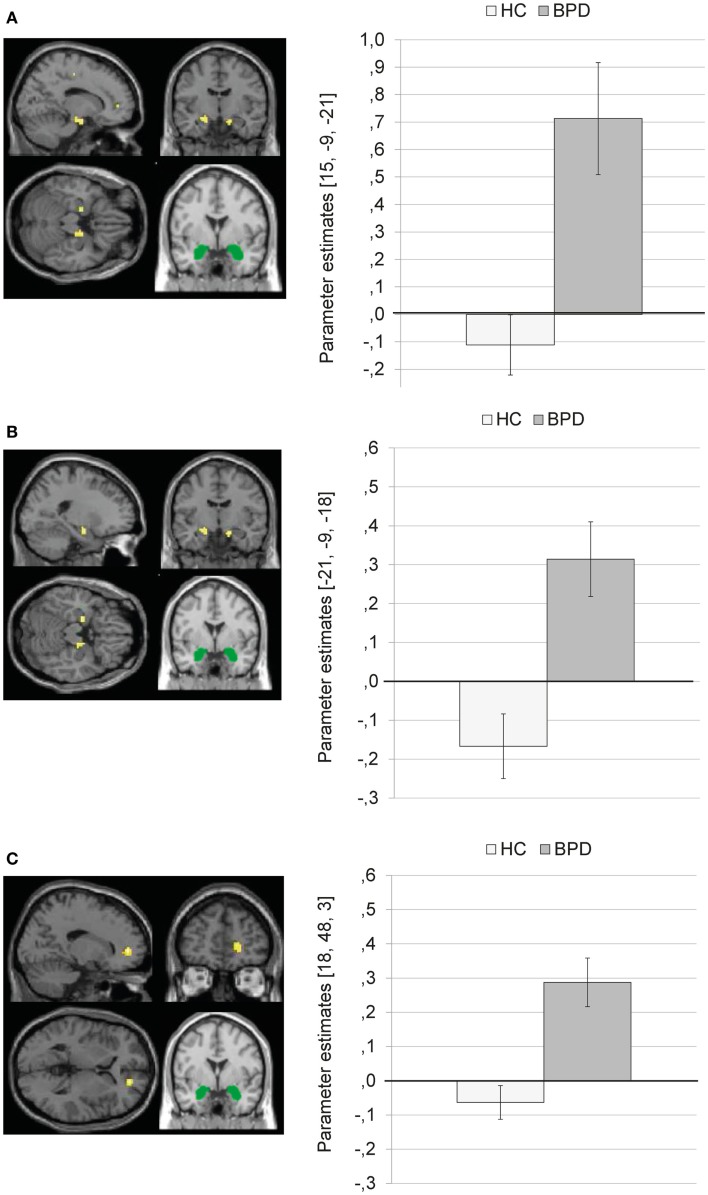
**Means ± standard errors of the mean (SEM) of parameter estimates for stronger connectivity of the bilateral amygdala seed (depicted in green) in borderline personality disorder (BPD) than in healthy controls (HC) during presentation of negative distractors**. **(A)** Amygdala connectivity with right parahippocampal gyrus (BA34, MNI: 15, −9, −21). **(B)** Amygdala connectivity with left (para)hippocampus (MNI: −21, −9, −18). **(C)** Amygdala connectivity with right medial frontal gyrus (BA10, MNI: 18, 48, 3).

Borderline personality disorder patients showed a stronger coupling of the amygdala with clusters in the right parahippocampal gyrus (BA34) (Figure 4A) and left parahippocampal gyrus/hippocampus (Figure 4B) than HC. In BPD patients, positive amygdala connectivity with these brain areas was observed, while HC showed negative amygdala connectivity with these regions during presentation of negative distractors (see Figure 4).

The SVC with the dmPFC mask revealed a stronger coupling of the amygdala with a cluster in the right medial frontal gyrus (BA10) in BPD than in HC (see Table S3 in Supplementary Material). Figure 4C shows that there was positive amygdala connectivity with right medial frontal gyrus in BPD patients, while HC showed negative amygdala connectivity with this region.

The SVC with the dlPFC mask revealed no significant clusters.

There were no significant results for the T contrast HC > BPD.

### Dorsal anterior cingulate (dACC) connectivity

#### Main effects and interaction effect (2 × 2 Full Factorial Model)

Results of the main effects and interaction effects of the 2 × 2 Full Factorial Model for dACC connectivity can be found in Table S4 in Supplementary Material.

##### Main effect of valence

The 2 × 2 Full Factorial Model revealed a significant main effect of valence for dACC connectivity with bilateral lingual gyrus (BA19), bilateral fusiform gyrus, right posterior cingulate/cingulate gyrus, and bilateral middle/superior temporal gyrus.

The coupling of dACC with these brain regions was significantly weaker during presentation of negative distractors than during presentation of neutral distractors (see Table S5 in Supplementary Material). The T contrast negative > neutral distractors revealed no significant clusters (see Table S5 in Supplementary Material).

##### Main effect of group

**Figure 5:**
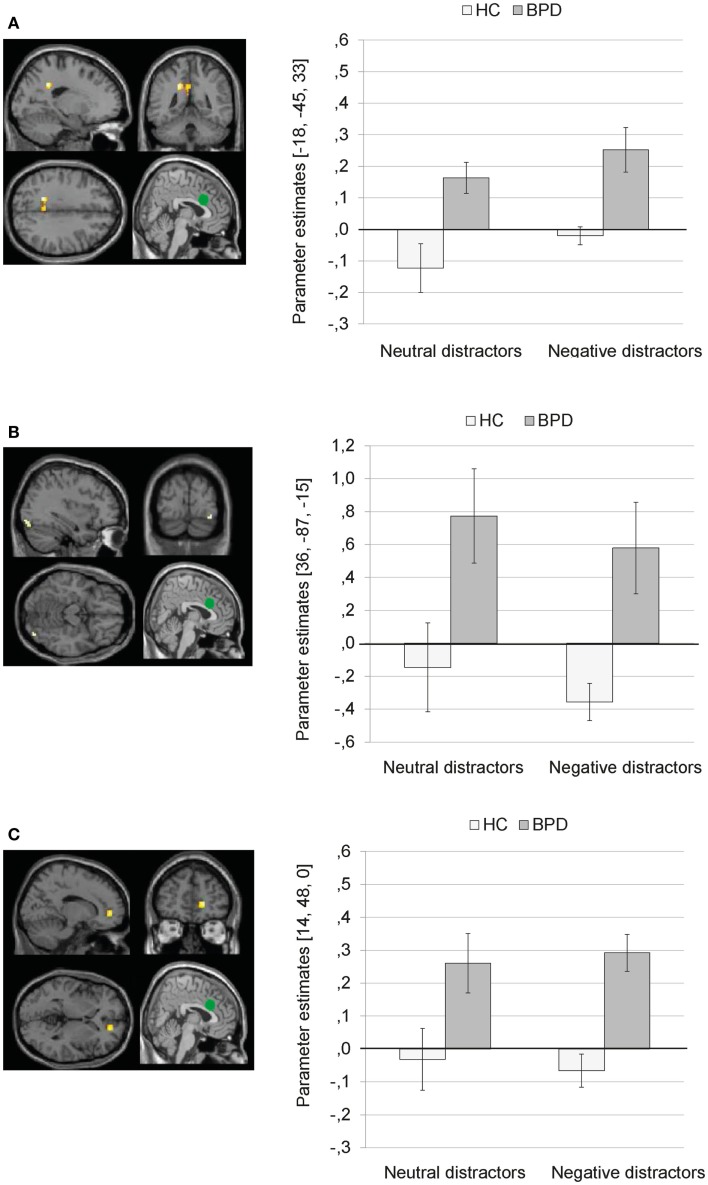
**Means ± standard errors of the mean (SEM) of parameter estimates for connectivity of the bilateral dorsal anterior cingulate (dACC) seed (depicted in green) during presentation of neutral distractors and negative distractors in patients with borderline personality disorder (BPD) and healthy controls (HC) (main effect of group of the 2 × 2 Full Factorial Model)**. **(A)** dACC connectivity with left precuneus (MNI: −18, −45, 33). **(B)** dACC connectivity with right inferior occipital gyrus (MNI: 36, −87, −15). **(C)** dACC connectivity with right ACC (14, 48, 0).

There was a significant main effect of group on dACC connectivity with a cluster comprising left precuneus and posterior cingulate (BA31), as well as clusters in the right inferior occipital gyrus, and right ACC (BA32). Figure 5 illustrates that BPD patients showed positive dACC connectivity with these regions, while HC showed negative dACC connectivity with these brain areas during both conditions of the EWMT.

##### Interaction effect

**Figure 6:**
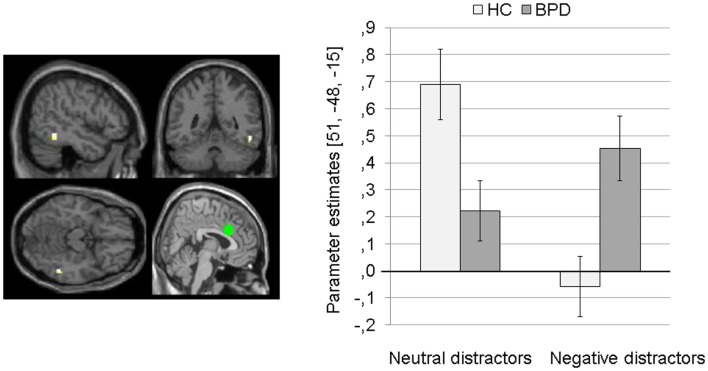
**Means ± standard errors of the mean (SEM) of parameter estimates for connectivity of the bilateral dorsal anterior cingulate (dACC) seed (depicted in green) with right superior temporal gyrus (MNI: 51, −48, −15) revealed by the interaction effect of group by valence of the 2 × 2 Full Factorial Model for dACC connectivity in patients with borderline personality disorder (BPD) and healthy controls (HC)**.

The 2 × 2 Full Factorial Model further revealed a significant interaction effect of valence by group on amygdala connectivity with right superior temporal gyrus. During both EWMT conditions, BPD patients showed positive dACC connectivity with right superior temporal gyrus (most prominently during presentation of negative distractors). HC showed positive dACC connectivity during presentation of neutral distractors und negative dACC connectivity with this region during negative distractors (see Figure 6).

#### Independent *t*-test for group differences during negative distractors

Complete results of the independent *t*-test for dACC connectivity during presentation of negative distractors can be found in Table S6 in Supplementary Material.

Compared to HC, BPD patients showed a stronger coupling of the dACC with right medial frontal gyrus, left inferior parietal lobule, left precentral gyrus, left insula, left posterior cingulate, left inferior/middle occipital gyrus, left paracentral lobule, left superior temporal gyrus, and left precentral gyrus.

**Figure 7:**
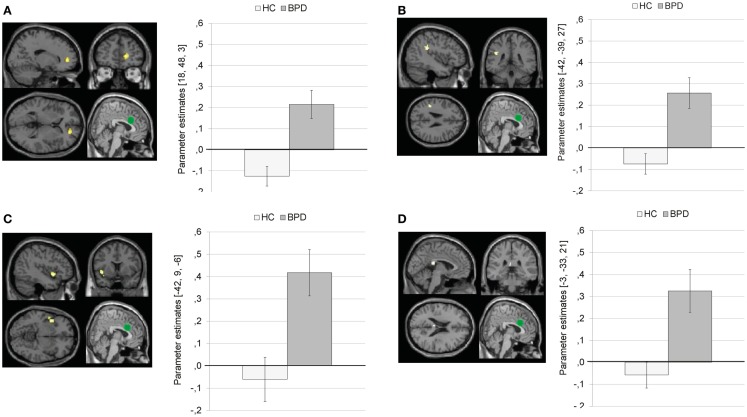
**Means ± standard errors of the mean (SEM) of parameter estimates for stronger connectivity of the bilateral dorsal anterior cingulate (dACC) seed (depicted in green) during presentation of negative distractors in patients with borderline personality disorder (BPD) than in healthy controls (HC)**. **(A)** dACC connectivity with right medial frontal gyrus (MNI: 18, 48, 3). **(B)** dACC connectivity with left inferior parietal lobule (MNI: −42, −39, 27). **(C)** dACC connectivity with left insula (BA13, MNI: −42, 9, −6). **(D)** Connectivity with left posterior cingulate (BA23; MNI: −3, −33, 21).

Figure 7 illustrates that BPD patients demonstrated positive dACC connectivity with right medial frontal gyrus (BA10), left inferior parietal lobule, left insula, and left posterior cingulate, while HC showed negative connectivity between these regions.

There were no significant results for the T contrast HC > BPD.

### Regression analyses

Results of the whole-brain regression analysis for reaction times as regressor of interest for amygdala connectivity during presentation of negative distractors are presented in Table S7 in Supplementary Material.

**Figure 8:**
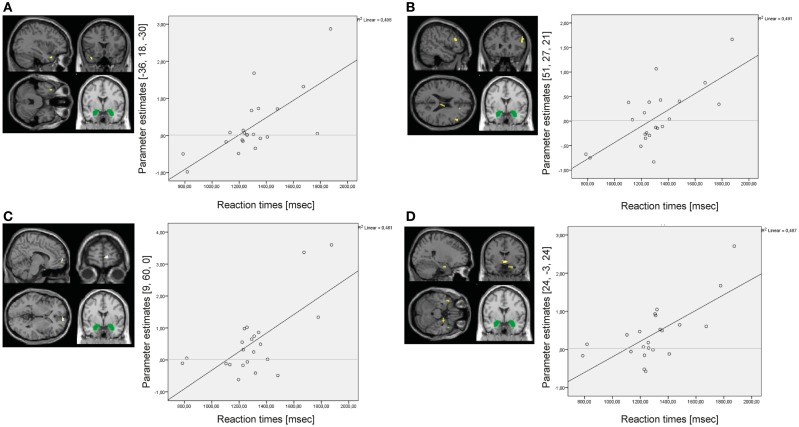
**Results of the whole-brain regression analysis with reaction times as regressor of interest for connectivity of the bilateral amygdala seed (depicted in green) during presentation of negative distractors in the group of borderline personality disorder (BPD) patients**. **(A)** Regression for amygdala connectivity with left superior temporal gyrus (MNI: −36, 18, −30). **(B)** Regression for amygdala connectivity with right middle frontal gyrus (MNI: 51, 27, 21). **(C)** Regression for amygdala connectivity with right medial frontal gyrus (MNI: 9, 60, 0). **(D)** Regression for amygdala connectivity with right parahippocampal gyrus/hippocampus (MNI: 24, −3, 24).

In the BPD group, reaction times positively predicted amygdala connectivity with left superior temporal gyrus (BA38), right middle frontal gyrus (BA46), right medial frontal gyrus (BA10), and right parahippocampal gyrus/hippocampus (see Figure 8). There were no significant results of the same regression analysis in the HC group (at *p* < 0.001, *k* ≥ 10, *Z* > 3.1).

**Figure 9:**
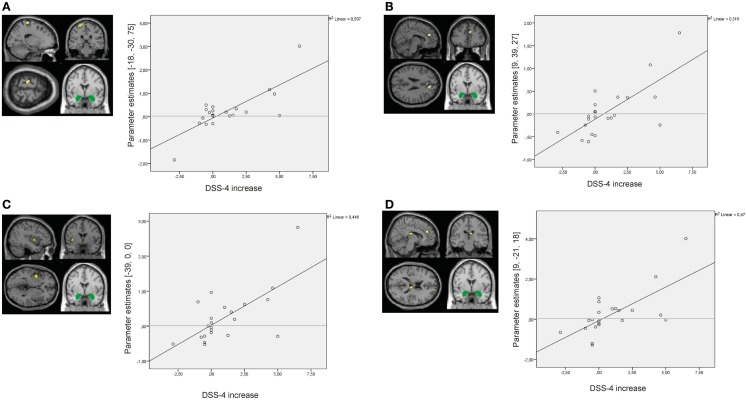
**Results of the whole-brain regression analysis with self-reported increase of dissociation (DSS-4 scores) as regressor of interest for connectivity of the bilateral amygdala seed (depicted in green) during presentation of negative distractors in the group of borderline personality disorder (BPD) patients**. **(A)** Regression for amygdala connectivity with left precentral gyrus (MNI: −18, −30, 75). **(B)** Regression for amygdala connectivity with right anterior cingulate cortex (9, 39, 27). **(C)** Regression for amygdala connectivity with left insula (−39, 0, 0). **(D)** Regression for amygdala connectivity with right thalamus (9, −21, 18). DSS-4, Dissociation Stress Scale 4.

Results of the whole-brain regression analysis with self-reported increase of state dissociation (mean increase of DSS-4 scores) for amygdala connectivity during presentation of negative distractors in the BPD group are presented in Table S8 in Supplementary Material. Self-reported state dissociation positively predicted amygdala connectivity with clusters in the left precentral gyrus (BA4), right ACC (BA32), right thalamus, and left insula (BA13) (see Figure 9).

## Discussion

We used PPI analysis to investigate functional connectivity during performance of an EWMT in 22 unmedicated female BPD patients with a history of interpersonal trauma and 22 healthy women (HC). The bilateral amygdala as well as bilateral dACC was defined as seed regions of interest. The main results were:

- Reduced amygdala connectivity with clusters in the left dlPFC (superior frontal gyrus) and left ventrolateral PFC (inferior frontal gyrus) during emotional distraction in the whole group.
- Stronger positive amygdala connectivity with bilateral (para-)hippocampus as well as stronger positive dACC connectivity with left insula, posterior cingulate, superior temporal gyrus, and occipital gyrus in BPD patients during emotional distraction. Compared to HC, BPD patients further showed a stronger coupling of both the amygdala and dACC seed with a cluster in the right dmPFC (medial frontal gyrus).
- Reaction times positively predicted amygdala connectivity with right dorsomedial and dorsolateral PFC and right (para)hippocampus during emotional distraction in the BPD group.
- Self-reported state dissociation positively predicted amygdala connectivity with right ACC, left precentral gyrus, left insula, and right thalamus during emotional distraction in patients.

These results are discussed per seed in the following.

### Amygdala connectivity

In the whole group, a reduced coupling of the amygdala with clusters in the left dlPFC (superior frontal gyrus) and left vlPFC (inferior frontal gyrus) as well as right caudate was observed, when negative (compared to neutral) IAPS pictures were presented during the delay interval of the working memory task. The inferior frontal gyrus, superior frontal gyrus, and caudate are parts of a prefrontal-striato-thalamo-cortical loop, which has been implicated in interference inhibition and basic working memory processes including the maintenance of information across a delay (Goldman-Rakic et al., 1992; McGaugh, 2004; Seger and Cincotta, 2005; Dolcos et al., 2006; Geier et al., 2009; Grahn et al., 2009; Aron et al., 2014). Our finding suggests a reduced information exchange between the amygdala (i.e., a brain region implicated in emotion processing) and regions involved in working memory maintenance, possibly reflecting a disruptive effect of emotional distraction in the whole group.

Importantly, there were significant group differences in amygdala connectivity during emotional distraction: Compared to HC, BPD patients showed a stronger coupling of the amygdala with right dmPFC (medial frontal gyrus). Moreover, reaction times positively predicted amygdala connectivity with right dmPFC (medial frontal gyrus) and right dlPFC (middle frontal gyrus) during emotional interference in the BPD group. This means, a stronger positive coupling of the amygdala with dorsomedial and dorsolateral prefrontal regions was associated with more working memory impairments after emotional distraction in BPD patients.

While patients showed positive amygdala with right dmPFC and left dlPFC, HC showed negative amygdala connectivity (suggesting inhibitory interactions) with these regions. In line with the latter finding, negative amygdala connectivity with dorsal prefrontal regions was also observed in previous fMRI studies investigating the neural correlates of emotional distraction in non-clinical samples (Mitchell et al., 2008; Anticevic et al., 2010). Interestingly, activity in prefrontal regions commonly engaged during working memory tasks (Miller, 2000; Barbey et al., 2013) has also been associated with cognitive emotion regulation (Schweizer et al., 2013). Parts of the dorsomedial and dorsolateral PFC, ventrolateral PFC, and anterior cingulate were found to be more active during emotion downregulation (e.g., reappraisal) in healthy individuals (Bush et al., 2000; Phan et al., 2002; Phillips et al., 2003; Phan et al., 2005; Ochsner and Gross, 2007; Etkin et al., 2011; Paret et al., 2011; Ochsner et al., 2012; Schweizer et al., 2013). In BPD patients, diminished activity in the dlPFC, vlPFC (Koenigsberg et al., 2009a), ACC (Lang et al., 2012), and OFC was found during cognitive reappraisal. Moreover, better emotion downregulation was related to a stronger negative coupling between amygdala and dorsomedial/dorsolateral PFC (Lee et al., 2012) and ventromedial/ventrolateral PFC (Johnstone et al., 2007; Townsend et al., 2013) in healthy individuals, while patients with affective disorders showed positive amygdala-prefrontal connectivity (Johnstone et al., 2007; Townsend et al., 2013). In healthy individuals, the recruitment of dorsal prefrontal regions during a working memory task may – either directly or indirectly via other brain regions – suppress amygdala signals during emotional distraction (Anticevic et al., 2010). PPI does not allow causal conclusions about the direction of interactions (i.e., whether the observed interactions reflect “bottom–up” or “top–down” directed mechanisms). Future studies should, therefore, use approaches like dynamic Causal Modeling to explicitly test causal models of a pre-defined network of assumed interactions.

In our present study, we further observed a stronger coupling of the amygdala with bilateral (para)hippocampus during emotional distraction in BPD patients than in HC. A stronger coupling of the amygdala with right (para)hippocampus was associated with longer reaction times in the patient group. The hippocampus and parts of the parahippocampal gyrus play an important role in memory encoding and retrieval (Squire and Zola-Morgan, 1991). The amygdala appears to modulate encoding and storage of emotional memories in the hippocampal formation, while the hippocampus is assumed to form representations of the emotional significance of events, thereby modulating amygdala response to these stimuli (Knight et al., 2004; McGaugh, 2004; Phelps, 2004; Richter-Levin and Akirav, 2000; Banich et al., 2009; Dolcos et al., 2012). Stronger activation and co-activation in the amygdala, hippocampus, and parahippocampal gyrus has been associated with enhancing effects of emotions on long-term episodic memory (Smith et al., 2006; Hahn et al., 2010; Dolcos et al., 2012), as well as fear conditioning (Tzschoppe et al., 2014). There is evidence that stress leads to enhanced memory retrieval in patients with BPD and patients with PTSD (Wingenfeld et al., 2012; Wingenfeld and Wolf, 2014). In previous experimental research, BPD patients further showed a memory recall bias for (negative) emotional information (e.g., see Baer et al., 2012; Winter et al., 2014). In the context of these earlier studies, our present finding may reflect an enhanced processing and encoding of task-irrelevant – but potentially self-relevant – emotional social information, which may interfere with cognitive performance during the working memory task in BPD patients.

During presentation of normative neutral distractors (neutral interpersonal IAPS pictures), BPD patients showed positive amygdala connectivity with right lingual gyrus, while HC showed negative connectivity between the regions. The lingual gyrus has been implicated in the encoding and retrieval of visual information including complex scenes and faces (Machielsen et al., 2000; Geier et al., 2009; Meng et al., 2012). Recently, increased activity in lingual gyrus was reported in BPD patients during the anticipation of negative pictures (Scherpiet et al., 2014). Our finding, therefore, suggests enhanced processing and enhanced affective evaluation of normative neutral social stimuli in patients with BPD. This will be discussed in more detail below.

### Self-reported in increase of dissociation

Interestingly, a stronger coupling of the amygdala with frontal regions (right ACC, left precentral gyrus), left insula, and right thalamus during emotional distraction was related to a stronger increase of dissociation (during the experiment) in the BPD group. In the same sample of BPD patients, trait dissociation positively predicted amygdala connectivity with right dlPFC during resting state (Krause-Utz et al., 2014c). Our findings suggest that dissociative states may modulate amygdala activity and connectivity during emotional challenge in patients with BPD. Dissociative states have been discussed as a regulatory strategy to cope with overwhelming emotional arousal in the face of traumatic situations or reminders (Lanius et al., 2010; Wolf et al., 2012). Further neuroimaging studies are needed to gain more insight into the neurobiological mechanisms possibly underlying this complex phenomenon. In particular, it remains an interesting topic for future studies to investigate the impact of dissociation on the neural correlates of other memory processes apart from working memory (e.g., episodic memory formation and retrieval) in BPD.

### Dorsal anterior cingulate connectivity

During emotional distraction, BPD patients did not only show a stronger coupling of the amygdala seed but also of the dACC seed with a cluster in the right dmPFC (medial frontal gyrus), which may be related to increased attention to negative interpersonal pictures (Ramnani and Owen, 2004; Reynolds et al., 2006; Burgess et al., 2007; Koechlin and Hyafil, 2007). In addition, BPD patients demonstrated stronger positive dACC connectivity with insula, posterior cingulate, precuneus, and superior temporal gyrus – brain areas involved in salience detection and attention (Bigler et al., 2007; Radua et al., 2010). Positive dACC connectivity with superior temporal gyrus was increased in BPD patients, but decreased (in terms of negative connectivity) in HC during emotional distraction. The superior temporal gyrus is assumed to play an important role in social cognition processes including the perception of facial stimuli (Bigler et al., 2007; Radua et al., 2010).

Group differences in dACC connectivity were not only observed for presentation of negative distractors but also for neutral distractors. BPD patients showed stronger positive dACC connectivity with left posterior cingulate and precuneus during both EWMT conditions, while HC showed negative connectivity between these regions. The posterior cingulate has been implicated in various functions including attention regulation, working memory, episodic memory, and monitoring of arousal states, although its precise role remains unknown (see e.g., Raichle et al., 2001; Greicius et al., 2003; Menon and Uddin, 2010; Leech and Sharp, 2014). Activity in the posterior cingulate and precuneus has further been associated with self-referential processing (e.g., rumination, self-reflection) (Raichle et al., 2001; Greicius et al., 2003; Menon and Uddin, 2010; Menon, 2011). Previous research suggests that healthy individuals commonly show negative correlations between activity in the dACC (being part of a frontoparietal network usually activated during cognitive tasks) and posterior cingulate cortex (being a central node of the default mode network usually activated during rest) (Fox et al., 2005; Buckner and Vincent, 2007; Sridharan et al., 2008; Neumann et al., 2010; Leech and Sharp, 2014). A flexible modulation between intrinsic connectivity networks is assumed to be crucial for cognitive efficiency, although the nature of interactions between these brain networks is not yet understood (Buckner and Vincent, 2007; Berman et al., 2011; Liddle et al., 2011; van Wingen et al., 2013; Leech and Sharp, 2014). Previous studies in BPD have provided evidence for imbalanced inter-network connectivity during resting state (Wolf et al., 2011; Doll et al., 2013) and pain processing (Kluetsch et al., 2012) in patients with BPD. For example, diminished negative correlations between the dACC and the left posterior cingulate were observed during resting state in the same sample of interpersonally traumatized BPD patients who participated in our EWMT (Krause-Utz et al., 2014c).

Interpersonal disturbances, including difficulties in developing trust in others, a hypersensitivity to social rejection, feelings of being socially excluded even in apparently neutral situations, and a tendency to interpret normative neutral stimuli as more negatively, are important core features of BPD (Donegan et al., 2003; Koenigsberg et al., 2009b; Frick et al., 2012; Lis and Bohus, 2013; Mier et al., 2013; Roepke et al., 2013; Krause-Utz et al., 2014a). A stronger emotional involvement while processing social stimuli may hinder social-cognitive processing in patients with BPD (Mier et al., 2013). Previous fMRI studies in BPD patients further found increased activity in medial PFC during experimentally induced situations of social exclusion (Ruocco et al., 2010; Domsalla et al., 2013) but also during situations of social inclusion (Domsalla et al., 2013). In the context of this previous research, our present findings suggest an enhanced attention to – both negative and normative neutral – social information, which may elicit enhanced self-referential processing (e.g., retrieval of negative memories of interpersonal events) in patients with BPD.

To our knowledge, this is the first study investigating amygdala and dACC connectivity during performance of the EWM paradigm in unmedicated BPD patients with a history of interpersonal trauma compared to HC. Some limitations need to be addressed. First, we did not manipulate the cognitive load of our working memory task using sets of 3 × 3 items, which represents a moderate difficulty. The strength of the coupling between amygdala and dorsal prefrontal regions (e.g., dlPFC) may be dependent on the cognitive load of the task (Iordan et al., 2013). Moreover, the social dimension of distractors (e.g., using interpersonal scenes versus objects as distractors) may modulate amygdala connectivity (Britton et al., 2006). Second, we used PPI to investigate our hypothesis-driven research questions. By restricting our analysis to *a priori* defined seeds, our results are inherently limited to the connections of these seed with “coupled” areas. Data-driven methods such as ICA have the potential to analyze fMRI data in a more exploratory way. Moreover, as stated above, PPI does not allow causal conclusions about the direction of interactions and tend to lack power for event-related designs (O’Reilly et al., 2012). All patients included in our study reported a history of complex and severe interpersonal trauma and some patients met diagnosis for comorbid anxiety disorders (e.g., PTSD), which is highly prevalent in BPD (Bremner, 2006; Leichsenring et al., 2011). Therefore, our findings may be related to interpersonal trauma *per se* (Dannlowski et al., 2012; Herringa et al., 2013; Teicher and Samson, 2013; van der Werff et al., 2013; Elton et al., 2014) or to comorbid PTSD (Gilboa et al., 2004; Bluhm et al., 2009; Lanius et al., 2010b; Daniels et al., 2011; Rabinak et al., 2011; Sripada et al., 2012; Jin et al., 2013; Nooner et al., 2013; Stevens et al., 2013; Brown et al., 2014).

All in all, our findings suggest a disrupted information exchange between the amygdala (a brain region critically involved in emotion processing) and brain regions involved in working memory maintenance and interference inhibition during emotional distraction. Stronger amygdala and dACC connectivity with brain regions involved in salience detection, social cognition, and autobiographical memory retrieval may reflect difficulties in shifting attention away from task-irrelevant – but possibly self-relevant – social information and increased self-referential processes in patients with BPD.

## Conflict of Interest Statement

None of the authors declare biomedical financial interests or potential conflicts of interest. Investigator A. Krause-Utz was funded by a Ph.D. doctoral stipend of the SFB636 by the German Research Foundation. Investigator B. M. Elzinga was funded by a VIDI grant by the Netherlands Organisation for Scientific Research (grant number 016·085·353).

## Supplementary Material

The Supplementary Material for this article can be found online at http://www.frontiersin.org/Journal/10.3389/fnhum.2014.00848/abstract
