# Supplementary material for: Amygdala and Dorsal Anterior Cingulate Connectivity during an Emotional Working Memory Task in Borderline Personality Disorder Patients with Interpersonal Trauma History
Source: Front Hum Neurosci. 2014 Oct 28;8:848. doi: 10.3389/fnhum.2014.00848 (PMC4211399; doi:10.3389/fnhum.2014.00848)
Supplement: Supplementary file 1 [file Table_1.PDF]

Table S1: Results of the main effects and interaction effects of the 2x2 Full Factorial Model for task-related bilateral amygdala connectivity

F Contrast	Brain region of coactivation: Label (Brodman area)	Lobe	Cluster size	Peak voxel coordinates (MNI: X, Y, Z)	F value	Z value	p value
Main effect of Valence	Lingual Gyrus<br>Fusiform Gyrus (BA19)<br>Lingual Gyrus (BA18)	Occipital Lobe<br>Temporal Lobe	398	-9, -81, -3<br>24, -66, -12<br>-24, -78, -9	36.29<br>19.58<br>17.56	5.35<br>4.02<br>3.81	p<0.001
	Parahippocampal Gyrus (BA19)<br>Parahippocampal Gyrus (BA36) /<br>Fusiform Gyrus	Limbic Lobe<br>Limbic Lobe<br>Anterior Lobe	177	-24, -48, -9<br>-27, -36, -18<br>-36, -51, -24	29.21<br>19.44<br>15.21	4.86<br>4.01<br>3.55	p<0.001
	Inferior Frontal Gyrus (BA47)	Frontal Lobe	39	-30, 30, -18	23.43	4.38	p<0.001
	Fusiform Gyrus (BA20)	Temporal Lobe	36	33, -39, -21	23.13	4.36	p<0.001
	Posterior Cingulate (BA29)	Limbic Lobe	17	9, -48, 18	22.25	4.28	p<0.001
	Middle Temporal Gyrus (BA21)	Temporal Lobe	16	51, -12, -18	19.21	3.99	p<0.001
	Posterior Cingulate (BA23)	Limbic Lobe	29	3, -36, 27	18.06	3.87	p<0.001
	Caudate	Sub-lobar	11	9, 3, 18	18.02	3.86	p<0.001
	Hippocampus	Limbic Lobe	20	-18, -6, -21	16.42	3.69	p<0.001
	Superior Frontal Gyrus (BA9)*	Frontal Lobe*	11*	-21, 43, 45*	12.88*	3.26*	p<0.01*
Main effect of Group	Lingual Gyrus	Occipital Lobe	10	3, -84, -15	12.83	3.25	p<0.001
Interaction effect	No significant clusters at p<0.001 (k≥10, Z<3.1)						

Note: Clusters were determined using a significant threshold of *p*<0.001 uncorrected at a voxel-wise whole-brain level. Clusters exceeding a Z-value of >3.1 and a cluster size of k≥10 contiguous voxels are presented. Small volume corrections (SVC) were applied for dorsolateral as well as dorsomedial prefrontal regions (using anatomical masks based on the Automatic Anatomical Labeling software as provided in SPM8). Clusters determined by SVC are indicated by an asterisk (*)
