# Supplementary material for: Amygdala and Dorsal Anterior Cingulate Connectivity during an Emotional Working Memory Task in Borderline Personality Disorder Patients with Interpersonal Trauma History
Source: Front Hum Neurosci. 2014 Oct 28;8:848. doi: 10.3389/fnhum.2014.00848 (PMC4211399; doi:10.3389/fnhum.2014.00848)
Supplement: Supplementary file 2 [file Table_2.PDF]

Table S2: T contrasts for neutral > negative distractors and negative > neutral distractors within the 2x2 Full Factorial Model of task-related amygdala connectivity

T Contrast	Brain region of coactivation: Label (Brodman area)	Lobe	Cluster size	Peak voxel coordinates (MNI: X, Y, Z)	T value	Z value	p value
Neutral distractors > negative distractors (in the whole group)	Lingual Gyrus<br>Fusiform Gyrus (BA19)<br>Lingual Gyrus (BA18)	Occipital Lobe<br>Temporal Lobe	550	-9, -81, -3<br>24, -66, -12<br>-24, -78, -9	6.02<br>4.43<br>4.19	5.48<br>4.18<br>3.98	p<0.001
	Parahippocampal Gyrus (BA19)<br>Parahippocampal Gyrus (BA36)/ Fusiform Gyrus	Limbic Lobe<br>Limbic Lobe	227	-24, -48, -9<br>-27, -36, -18	5.40<br>4.41	4.99<br>4.17	p<0.001
	Inferior Frontal Gyrus (BA47)	Frontal Lobe	55	-30, 30, -18	4.84	4.53	p<0.001
	Fusiform Gyrus (BA20)	Temporal Lobe	54	33, -39, -21	4.81	4.51	p<0.001
	Posterior Cingulate (BA29)	Limbic Lobe	21	9, -48, 18	4.72	4.43	p<0.001
	Precuneus / Cingulate Gyrus	Limbic Lobe	16	9, -48, 42	4.22	4.01	p<0.001
	Middle Temporal Gyrus (BA21)	Temporal Lobe	30	51, -12, -18	4.38	4.15	p<0.001
	Cingulate Gyrus	Limbic Lobe	12	18, -54, 27	4.36	4.13	p<0.001
	Hippocampus	Limbic Lobe	59	-18, -6, -21<br>-24, -12, -18	4.05<br>4.04	3.86<br>3.85	p<0.001
	Superior Temporal Gyrus (BA38)	Temporal Lobe	59	-39, 3, -21	3.64	3.50	p<0.001
	Thalamus	Sub-lobar	24	-6, -30, 3	3.28	3.18	p<0.001
	Caudate	Sub-lobar	15	9, 3, 18	4.24	4.03	p<0.001
	Posterior Cingulate (BA23)	Limbic Lobe	38	3, -36, 27	4.25	4.03	p<0.001
	Cingulate Gyrus	Limbic Lobe	23	12, -30, 39	3.57	3.44	p<0.001
	Superior Frontal Gyrus (BA9)*	Frontal Lobe*	15*	-21, 42, 45*	3.37*	3.25*	p<0.01*
Negative > neutral distractors (whole group)	No significant clusters at p<0.001 (k≥10, Z>3.1)						

Note: Clusters were determined using a significant threshold of *p*<0.001 uncorrected at a voxel-wise whole-brain level. Clusters exceeding a Z-value of >3.1 and a cluster size of k≥10 contiguous voxels are presented. Small volume corrections (SVC) were applied for dorsolateral as well as dorsomedial prefrontal regions (using anatomical masks based on the Automatic Anatomical Labeling software as provided in SPM8). Clusters determined by SVC are indicated by an asterisk (*)
