# Supplementary material for: Amygdala and Dorsal Anterior Cingulate Connectivity during an Emotional Working Memory Task in Borderline Personality Disorder Patients with Interpersonal Trauma History
Source: Front Hum Neurosci. 2014 Oct 28;8:848. doi: 10.3389/fnhum.2014.00848 (PMC4211399; doi:10.3389/fnhum.2014.00848)
Supplement: Supplementary file 3 [file Table_3.PDF]

Table S3: Results of the between-group differences for bilateral amygdala connectivity during emotional distraction in Borderline Personality Disorder (BPD) patients and healthy controls (HC)

**T Contrast**	**Brain region of coactivation: Label (Brodman area)**	**Lobe**	**Cluster size**	**Peak voxel coordinates (MNI: X, Y, Z)**	**T-value**	**Z-value**	**Significance level (uncorrected)**
BPD>HC	Parahippocampal Gyrus (BA34)	Limbic Lobe	20	15, -9, -21	4.02	3.67	p<0.001
	Parahippocampal Gyrus/ Hippocampus	Limbic Lobe	19	-21, -9, -18	3.94	3.61	p<0.001
	Medial Frontal Gyrus (BA10)*	Frontal Lobe*	23*	18, 48, 3*	4.50*	4.04*	p<0.01*
HC>BPD	No significant clusters at p<0.001 (k>10, Z>3.1)						

Note: Clusters were determined using a significant threshold of $p<0.001$ uncorrected at a voxel-wise whole-brain level. Clusters exceeding a Z-value of >3.1 and a cluster size of k≥10 contiguous voxels are presented. Small volume corrections (SVC) were applied for dorsolateral as well as dorsomedial prefrontal regions (using anatomical masks based on the Automatic Anatomical Labeling software as provided in SPM8). Clusters determined by SVC are indicated by an asterisk (*)
