# Supplementary material for: Amygdala and Dorsal Anterior Cingulate Connectivity during an Emotional Working Memory Task in Borderline Personality Disorder Patients with Interpersonal Trauma History
Source: Front Hum Neurosci. 2014 Oct 28;8:848. doi: 10.3389/fnhum.2014.00848 (PMC4211399; doi:10.3389/fnhum.2014.00848)
Supplement: Supplementary file 4 [file Table_4.PDF]

Table S4: Results of the main effects and interaction effects of the 2x2 Full Factorial Model for task-related bilateral dorsal anterior cingulate seed connectivity

**Contrast**	**Brain region of coactivation: Label (Brodman area)**	**Lobe**	**Cluster size**	**Peak voxel coordinates (MNI: X, Y, Z)**	**F-value**	**Z-value**	**Significance level (uncorrected)**
Main effect of Valence	Lingual Gyrus (BA19)<br>Fusiform Gyrus<br>Lingual Gyrus	Occipital Lobe	653	-9, -81, -6<br>33, -39, -18<br>24, -69, -9	31.66<br>26.27<br>24.45	5.04<br>4.63<br>4.47	p<0.001
	Parahippocampal Gyrus (BA19)<br>Fusiform Gyrus	Limbic Lobe<br>Temporal Lobe	151	-24, -48, -9<br>-27, -39, -18	27.00<br>19.95	4.68<br>4.06	p<0.001
	Cingulate Gyrus	Limbic Lobe	11	9, 9, 36	19.10	3.97	p<0.001
	Middle Temporal Gyrus (BA39)	Temporal Lobe	22	-45, -78, 18	17.49	3.81	p<0.001
	Superior Temporal Gyrus (BA22)	Temporal Lobe	20	60, -54, 9	16.76	3.73	p<0.001
	Cingulate Gyrus (BA31)<br>Cingulate Gyrus (BA31)	Limbic Lobe	20	9, -30, 39<br>9, -39, 42	15.68<br>15.64	3.60<br>3.60	p<0.001
Main Effect of Group	Precuneus (BA31)<br>Posterior Cingulate (BA31)	Parietal Lobe<br>Limbic Lobe	37	-18, -45, 33<br>-3, -45, 33	22.95<br>14.62	4.34<br>3.48	p<0.001
	Anterior Cingulate (BA32)	Limbic Lobe	21	14, 48, 0	19.87	4.05	p<0.001
	Inferior Occipital Gyrus (BA18)	Occipital Lobe	10	36, -87, -15	14.58	3.47	p<0.001
Interaction effect	Middle/Superior Temporal Gyrus	Temporal Lobe	12	51, -48, -15	17.38	3.79	p<0.001

Note: Clusters were determined using a significant threshold of $p<0.001$ uncorrected at a voxel-wise whole-brain level. Clusters exceeding a Z-value of >3.1 and a cluster size of k≥10 contiguous voxels are presented.
