# Supplementary material for: Amygdala and Dorsal Anterior Cingulate Connectivity during an Emotional Working Memory Task in Borderline Personality Disorder Patients with Interpersonal Trauma History
Source: Front Hum Neurosci. 2014 Oct 28;8:848. doi: 10.3389/fnhum.2014.00848 (PMC4211399; doi:10.3389/fnhum.2014.00848)
Supplement: Supplementary file 5 [file Table_5.PDF]

Table S5: Results of T contrasts for neutral > negative distractors and negative > neutral distractors within the 2x2 Full Factorial Model of task-related bilateral dorsal anterior cingulate cortex (dACC) connectivity

**T Contrast**	**Brain region of coactivation: Label (Brodman area)**	**Lobe**	**Cluster size**	**Peak voxel coordinates (MNI: X, Y, Z)**	**T value**	**Z value**	**p value**
Neutral distractors > negative distractors (in the whole group)	Lingual Gyrus (BA19)<br>Fusiform Gyrus<br>Lingual Gyrus	Occipital Lobe	855	-9, -81, -6<br>33, -39, -18<br>24, -69, -9	5.63<br>5.13<br>4.94	5.17<br>4.77<br>4.62	p<0.001
	Parahippocampal Gyrus<br>Fusiform Gyrus	Limbic Lobe<br>Temporal Lobe	198	-24, -48, -9<br>-27, -39, -18	5.20<br>4.47	4.82<br>4.22	p<0.001
	Cingulate Gyrus	Limbic Lobe	15	9, 9, 36	4.37	4.14	p<0.001
	Cingulate Gyrus	Limbic Lobe	11	18, -54, 27	4.19	3.98	p<0.001
	Middle Temporal Gyrus	Temporal Lobe	37	-45, -78, 18	4.18	3.97	p<0.001
	Superior Temporal Gyrus	Temporal Lobe	30	60, -54, 9	4.09	3.90	p<0.001
	Cingulate Gyrus<br>Cingulate Gyrus	Limbic Lobe	39	9, -30, 39<br>9, -39, 42	3.96<br>3.96	3.78<br>3.78	p<0.001
	Posterior Cingulate	Limbic Lobe	13	12, -48, 15	3.88	3.71	p<0.001
Negative > neutral distractors (whole group)	No significant clusters at p<0.001 (k≥10, Z>3.1)						

Note: Clusters were determined using a significant threshold of *p*<0.001 uncorrected at a voxel-wise whole-brain level. Clusters exceeding a Z-value of >3.1 and a cluster size of k≥10 contiguous voxels are presented.
