# Supplementary material for: Amygdala and Dorsal Anterior Cingulate Connectivity during an Emotional Working Memory Task in Borderline Personality Disorder Patients with Interpersonal Trauma History
Source: Front Hum Neurosci. 2014 Oct 28;8:848. doi: 10.3389/fnhum.2014.00848 (PMC4211399; doi:10.3389/fnhum.2014.00848)
Supplement: Supplementary file 6 [file Table_6.PDF]

Table S6: Results of the between-group differences for bilateral dorsal anterior cingulate connectivity during emotional distraction in Borderline Personality Disorder (BPD) patients and healthy controls (HC)

T Contrast	Brain region of coactivation: Label (Brodman area)	Lobe	Cluster size	Peak voxel coordinates (MNI: X, Y, Z)	T-value	Z-value	Significance level (uncorrected)
BPD>HC	Medial Frontal Gyrus (BA10)	Frontal Lobe	23	18, 48, 3	5.89	5.00	p<0.001
	Inferior Parietal Lobule	Parietal Lobe	20	-42, -39, 27	4.30	3.89	p<0.001
	Precental Gyrus<br>Insula	Frontal Lobe<br>Sub-lobar	12	-48, -12, 48<br>-42, 9, -6	4.15<br>3.68	3.78<br>3.40	p<0.001
	Posterior Cingulate (BA23)	Limbic Lobe	10	-3, -33, 21	3.69	3.42	p<0.001
	Medial Frontal Gyrus (BA6)	Frontal Lobe	11	18, -3, 54	4.13	3.77	p<0.001
	Inferior Occipital Gyrus (BA18)<br><br>Middle Occipital Gyrus (BA19)	Occipital Lobe	14	-36, -90, -15<br>-42, -84, -15<br>-48, -84, -3	4.02<br>3.88<br>3.63	3.67<br>3.57<br>3.36	p<0.001
	Paracentral Lobule (BA5)<br>Cingulate Gyrus (BA31)	Frontal Lobe<br>Limbic Lobe	12	-12, -36, 54<br>-15, -30, 48	4.01<br>3.70	3.67<br>3.42	p<0.001
	Superior Temporal Gyrus (BA22)	Temporal Lobe	14	-54, 0, -6	3.78	3.48	p<0.001
	Precentral Gyrus (BA6)	Frontal Lobe	11	-60, -3, 12	3.69	3.41	p<0.001
HC>BPD	No significant clusters at p<0.01, k>10, Z>3.1						

Note: Clusters were determined using a significant threshold of *p*<0.001 uncorrected at a voxel-wise whole-brain level. Clusters exceeding a Z-value of >3.1 and a cluster size of k≥10 contiguous voxels are presented.
