# Supplementary material for: Amygdala and Dorsal Anterior Cingulate Connectivity during an Emotional Working Memory Task in Borderline Personality Disorder Patients with Interpersonal Trauma History
Source: Front Hum Neurosci. 2014 Oct 28;8:848. doi: 10.3389/fnhum.2014.00848 (PMC4211399; doi:10.3389/fnhum.2014.00848)
Supplement: Supplementary file 7 [file Table_7.PDF]

Table S7: Regression analysis: Reaction times as predictor of positive amygdala connectivity during presentation of negative distractors

**Group**	**Brain region of coactivation**	**Lobe**	**Cluster size**	**Peak voxel coordinates (MNI: X, Y, Z)**	**T value**	**Z value**	**p value**	**r**	**R^2**
BPD	Superior Temporal Gyrus (BA38)	Temporal Lobe	15	-36, 18, -30<br>-42, 12, -24	4.45<br>4.36	3.67<br>3.61	<0.001	.706	.498
	Middle Frontal Gyrus (BA46)	Frontal Lobe	18	51, 27, 21	4.40	3.63	<0.001	.701	.491
	Medial Frontal Gyrus (BA10)	Frontal Lobe	15	9, 60, 0	4.14	3.48	<0.001	.679	.461
	Parahippocampal Gyrus / Hippocampus	Limbic Lobe	9	24, -3, 24	4.36	3.61	<0.001	.698	.487
HC	No significant clusters at p<0.001 (k>10, Z>3.1)								

Note: BPD= group of Borderline Personality disorder patients; HC= healthy control group. Clusters were determined using a significant threshold of *p*<0.001 uncorrected at a voxel-wise whole-brain level. Clusters exceeding a Z-value of >3.1 and a cluster size of k≥10 contiguous voxels are presented.
