# Supplementary material for: Amygdala and Dorsal Anterior Cingulate Connectivity during an Emotional Working Memory Task in Borderline Personality Disorder Patients with Interpersonal Trauma History
Source: Front Hum Neurosci. 2014 Oct 28;8:848. doi: 10.3389/fnhum.2014.00848 (PMC4211399; doi:10.3389/fnhum.2014.00848)
Supplement: Supplementary file 8 [file Table_8.PDF]

Table S8: Regression Analysis: Self-reported increase of dissociation (DSS4 score) as predictor of positive amygdala connectivity during presentation of negative distractors in the group of Borderline Personality disorder (BPD) patients

**Brain region of coactivation**	**Lobe**	**Cluster size**	**Peak voxel coordinates (MNI: X, Y, Z)**	**T value**	**Z value**	**p value**	**r**	**R^2**
Precentral Gyrus (BA4)	Frontal Lobe	49	-18, -30, 75	5.44	4.21	<0.001	.773	.597
Anterior Cingulate (BA32)	Limbic Lobe	13	9, 39, 27	4.64	3.78	<0.001	.720	.518
Thalamus	Sub-Lobar	15	9, -21, 18	4.21	3.52	<0.001	.686	.470
Insula (BA13)	Sub-lobar	15	-39, 0, 0	4.01	3.40	<0.001	.668	.446

Note: DSS-4: Dissociation Stress Scale 4; Clusters were determined using a significant threshold of $p<0.001$ uncorrected at a voxel-wise whole-brain level. Clusters exceeding a Z-value of >3.1 and a cluster size of k≥10 contiguous voxels are presented.
